# Developing Independent Living Support for Older Adults Using Internet of Things and AI-Based Systems: Co-Design Study

**DOI:** 10.2196/54210

**Published:** 2024-10-24

**Authors:** Claire M Timon, Emma Heffernan, Sophia Kilcullen, Louise Hopper, Hyowon Lee, Pamela Gallagher, Alan F Smeaton, Kieran Moran, Pamela Hussey, Catriona Murphy

**Affiliations:** 1 School of Population Health, RCSI University of Medicine and Health Sciences, Dublin, D02DH60, Ireland; 2 School of Nursing, Psychotherapy and Community Health, Dublin City University, Dublin, Ireland; 3 Royal College of Physicians, Dublin, Ireland; 4 School of Psychology, Dublin City University, Dublin, Ireland; 5 School of Computing, Dublin City University, Dublin, Ireland; 6 Insight SFI Research Centre for Data Analytics, Dublin City University, Dublin, Ireland; 7 School of Health & Human Performance, Dublin City University, Dublin, Ireland; 8 Centre for eIntegrated Care, Dublin City University, Dublin, Ireland

**Keywords:** independent living, gerontology, geriatric, older adult, elderly, aging, Internet of Things, IoT, wearable electronic device, medical device, daily living activities, quality of life, QoL, artificial intelligence, AI, algorithm, predictive model, predictive analytics, predictive system, practical model

## Abstract

**Background:**

The number of older people with unmet health care and support needs is increasing substantially due to the challenges facing health care systems worldwide. There are potentially great benefits to using the Internet of Things coupled with artificial intelligence to support independent living and the measurement of health risks, thus improving quality of life for the older adult population. Taking a co-design approach has the potential to ensure that these technological solutions are developed to address specific user needs and requirements.

**Objective:**

The aim of this study was to investigate stakeholders’ perceptions of independent living and technology solutions, identify stakeholders’ suggestions on how technology could assist older adults to live independently, and explore the acceptability and usefulness of a prototype Internet of Things solution called the NEX system to support independent living for an older adult population.

**Methods:**

The development of the NEX system was carried out in 3 key phases with a strong focus on diverse stakeholder involvement. The initial predesign exploratory phase recruited 17 stakeholders, including older adults and family caregivers, using fictitious personas and scenarios to explore initial perceptions of independent living and technology solutions. The subsequent co-design and testing phase expanded this to include a comprehensive web-based survey completed by 380 stakeholders, encompassing older adults, family caregivers, health care professionals, and home care support staff. This phase also included prototype testing at home by 7 older adults to assess technology needs, requirements, and the initial acceptability of the system. Finally, in the postdesign phase, workshops were held between academic and industry partners to analyze data collected from the earlier stages and to discuss recommendations for the future development of the system.

**Results:**

The predesign phase revealed 3 broad themes: loneliness and technology, aging and technology, and adopting and using technology. The co-design phase highlighted key areas where technology could assist older adults to live independently: home security, falls and loneliness, remote monitoring by family members, and communication with clients. Prototype testing revealed that the acceptability aspects of the prototype varied across technology types. Ambient sensors and voice-activated assistants were described as the most acceptable technology by participants. Last, the postdesign analysis process highlighted that ambient sensors have the potential for automatic detection of activities of daily living, resulting in key recommendations for future developments and deployments in this area.

**Conclusions:**

This study demonstrates the significance of incorporating diverse stakeholder perspectives in developing solutions that support independent living. Additionally, it emphasizes the advantages of prototype testing in home environments, offering crucial insights into the real-world experiences of users interacting with technological solutions.

## Introduction

The world is witnessing a rapidly aging population. Population aging can be seen as one of the greatest successes of public health, as a longer life brings opportunities, not only for older people and their families, but also for societies as a whole [1]. The extent of these opportunities and contributions however depends heavily on one factor: healthy and positive aging [1]. Therefore, mechanisms that support healthy and positive aging are essential to ensure older people can enjoy physical and mental health and well-being to their full potential. Globally, health care policies including Ireland’s “Sláintecare” [2] are focusing on extending the ability of older people to continue to live independently at home. This entails maintaining quality of life as well as working to reduce the costs of an older person’s care. This presents a grand challenge to ensure that older adults receive adequate and individualized care and support to maintain their health, well-being, and safety whilst living independently [3].

The Internet of Things (IoT) and artificial intelligence (AI) offer significant benefits for supporting older adults in living independently, through enhanced health monitoring, improved home safety, and increased social connection, which in turn can improve health-related quality of life [4,5]. In this context, IoT refers to a network of physical devices that communicate and exchange data, while AI-based systems use algorithms and machine learning to process and analyze this data. Wearable IoT devices like smartwatches and fitness trackers monitor vital signs such as heart rate, blood pressure, and blood glucose levels, providing critical data to health care providers [6-8]. Smart home devices and AI-powered digital assistants can also mitigate risks by detecting hazards like fires and intruders, as well as alleviating loneliness and managing chronic conditions such as diabetes and hypertension [9-11]. Previous system-based approaches such as The HABITAT system [12] aim to support the independence of older adults by integrating technologies like radio frequency identification, wearable electronics, wireless sensor networks, and AI to enhance daily living environments. However, the deployment of these technologies comes with challenges, notably privacy and security risks that could expose older adults to cybercrimes and unauthorized access to sensitive health data [13,14]. Addressing these concerns is crucial to ensure the safe and accepted use of IoT technologies in this population.

The NEX system is an advanced IoT platform designed to support the independent living of older adults by monitoring their daily activities unobtrusively. It integrates various technologies such as smartwatches, voice-activated assistants, contact sensors, and smart plugs to gather comprehensive data on users’ activities and environments. This system uses AI and machine learning algorithms to analyze this data, enabling the detection and prediction of changes in the routines of older adults, known as activities of daily living (ADLs). The objective is to offer prompt and customized support to improve the quality of life and safety of older adults living at home. By unobtrusively monitoring for changes in routine behavior, such as a notable decrease in daily step count, the NEX system enables caregivers to respond quickly and provide necessary interventions. To address privacy and security concerns related to these technologies, the NEX system was designed to be highly customizable, giving users autonomy over the components they chose to use. Users were fully informed about the data each technology component collected and its purpose.

Despite the mounting evidence of the role of technology in supporting older adults to live independently at home [4,15,16], evidence in the literature suggests that end user’s acceptability and usability of technology are often neglected [17] and the literature calls for a shift in emphasis from focusing on product design to the user perspective. Co-design is essential in developing technology for older adults, as it engages end users directly in the design process to ensure that solutions align with their specific needs and preferences and enhances the relevance, usability, and acceptance of technology [18]. This research describes the comprehensive co-design approach used in the development of the NEX system, which integrates the perspectives and insights of multiple stakeholders [19]. By involving a diverse array of stakeholders including older adults, caregivers, and health care professionals, the project ensures that the system addresses the real-world needs and preferences of its end users. This participatory approach enhances the relevance and usability of the system in order to foster greater acceptance and effectiveness. Furthermore, the development process includes rigorous prototype testing in the homes of older adults, providing authentic insights into how the system functions in everyday home-based settings. This iterative process, characterized by real-world testing and stakeholder involvement, is fundamental to creating effective and sustainable health technology solutions.

## Methods

### Overview

**Figure 1.**
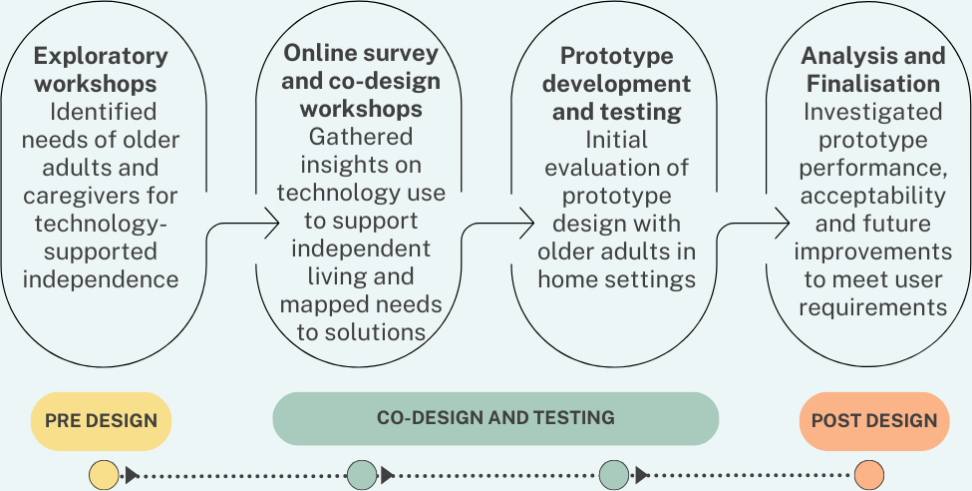
Co-design framework for the NEX system.

A user-centered design approach [20] was used to identify user needs and requirements. This co-design approach focuses on partnering with end users to design technology with them and not for them. This paper describes the use of a generative co-design framework for health care innovation [21] to inform the design of the NEX system. Figure 1 illustrates the adapted framework used to co-design the NEX system.

### Recruitment and Participant Profile

The recruitment process for the study involved multiple phases, starting with exploratory workshops aimed at older adults and family caregivers, who were recruited through Dublin City University Age Friendly network, social media, and local newspaper advertisements. The study’s co-design phase further expanded recruitment through a web-based survey targeting older adults, family caregivers, and health and social care professionals. This survey was advertised through social media and local council Age Friendly coordinators throughout Ireland. The final phase of recruitment occurred during the prototype development, where participants were those who had engaged in the web-based survey and expressed interest in further research participation. To ensure diverse participant recruitment a range of strategies were used ranging from web-based recruitment to recruiting and hosting one of the workshops at an older adults’ club in Dublin’s inner city. The recruitment for workshop and focus group activities ceased when thematic saturation was reached, indicating that sufficient data to robustly inform the design process was collected. Participants in the predesign and co-design workshops received a €20 (US $22) One4All voucher as compensation for their time and effort. Those who participated in the 10-week prototype testing phase were compensated with a €60 (US $66) One4All voucher.

The research engaged diverse participant groups across different phases. In the exploratory workshops, 17 participants including 15 older adults aged 60 years and older and 2 family caregivers participated. In the web-based survey, 380 respondents participated, comprising 235 older adults, 77 family caregivers, 47 health care professionals, and 21 homecare support staff. The majority were female (304/380, 80%) and well-educated with 58% (n=220) having completed tertiary education. The mean age of the cohort was 62.5 years (SD 11.5 years). Twenty-nine individuals including 15 older adults, 4 family caregivers, 4 health care professionals, and 6 home support workers participated in web-based workshops to discuss the utility and concerns regarding the various technology solutions proposed as part of the co-design phase. Lastly, 7 healthy older adults, aged 63 to 87 years, participated in a 10-week prototype testing of a system in their own homes. This sample size is based on population sizes from other published prototype testing studies in this area [22]. This group consisted of 5 women and 2 men, all living alone across Ireland, predominantly in urban areas. Living independently was a requirement for testing the prototype version of the system to ensure that most of the sensor data collected was attributable solely to the participant, not co-inhabitants. Due to COVID-19 restrictions limiting the movement of older adults, participants in the prototype testing self-installed the system with remote assistance from technical support over the phone. Future research will explore solutions for older adults living with caregivers or family. All described their health as “very good” or “excellent,” with most having chronic conditions. They were all regular smartphone users, and all had home broadband.

### Ethical Considerations

Ethical approval for the study was granted by the Dublin City University (DCU) Research Ethics Committee. The study adhered to strict data protection and ethical guidelines throughout its phases, from exploratory workshops to prototype testing. Participants’ contributions, whether in workshops or web-based surveys, were conducted with a focus on confidentiality and informed consent. Participation was voluntary, and oral and written information was provided to participants regarding the purpose of the study and how their data would be used in the research. The academic partners completed a data sharing agreement and conducted a data protection impact assessment to guarantee the protection and responsible use of participant data. While the industry partners had access to participants’ names and home addresses for technology installation purposes, they were restricted from accessing any health assessment data or any other personal information. To further ensure data security, all study-related data were securely stored on a designated drive at DCU. Ethical approval for conducting the predesign and co-design phases of this research was obtained from the DCU Research Ethics Committee under the reference DCUREC2019223, and for the prototype testing under the reference DCUREC2020180.

### Data Collection

Data collection methods varied across the study’s phases. In the predesign phase, exploratory workshops were conducted with potential end users (n=17), including older adults and family caregivers, to identify their needs and explore how technology could support their independence. These workshops were audio recorded and later transcribed to facilitate the exploration of the real-world needs and scenarios of users, setting a solid foundation for future technology design. Moving into the co-design and testing phase, a web-based survey (n=380 responses) was developed and disseminated using LimeSurvey platform to gain broader insights into older adults’ and stakeholders’ attitudes toward using technology for independent living. A user needs mapping exercise followed, linking these identified needs to potential technological solutions. Co-design workshops (n=29 participants) involving older adults, family caregivers, health care professionals, and home support workers facilitated in-depth discussions on these solutions, gathering feedback on their utility and acceptability. This phase also included the development of a prototype system that integrated various technologies, all tailored to the specific needs identified earlier.

The proposed prototype design consisted of contact sensors on entry and exit doors to the home and contact sensors on drawers and cupboards in the kitchen to detect activity around the house; smart plugs for kitchen appliances; 6-in-1 sensors to detect motion within rooms in the home alongside temperature and humidity; a Sony mWatch (smart watch device) as an alert system (call for assistance) and for measurement of sleep duration and step count; and an Amazon Echo Show 8 voice-activated assistant for entertainment or leisure use and reminder functionality. The prototype was subsequently tested in a real-world environment, with older adults (n=7) testing the prototype in their home environments for a period of 10 weeks. Participants also completed a process evaluation interview with a researcher via Zoom providing critical feedback on usability, acceptability, and technical performance, aiding in further refinement. In the postdesign phase, the feedback and data collected from the testing were analyzed to extract key insights, which were instrumental in refining the technology and finalizing the design, ensuring it aligned with the users’ needs and requirements effectively.

### Data Analysis

Quantitative survey responses were analyzed using descriptive statistics in SPSS (IBM Corp). Thematic analysis, as outlined by Braun and Clarke [23], is a qualitative research method used to systematically identify and analyze patterns or themes within data. This approach was applied to workshop and interview transcripts to uncover key themes and insights using NVIVO (Lumivero) qualitative data analysis software. The findings from this analysis were integral to the iterative design process, guiding the development of prototypes and informing the final design and functionality of the NEX system.

## Results

### Exploratory Workshop

Three broad themes emerged from the thematic analysis of the transcripts from the exploratory workshops based on the aims: loneliness and technology, aging and technology, and adopting and using technology (Table 1).

**Table 1.** Key themes from predesign workshops.

Theme	Key findings	Participant quotes
Loneliness and technology	Loneliness is a significant issue for older adults, particularly postretirement, and may lead to mental health concerns. Technology can potentially mitigate some aspects of loneliness by providing means of communication and maintaining social connections.	*If you could have some kind of a system, it is probably technology that could link a person who is isolated at home with some kind of community group or something.* [Family caregiver 01]
Aging and technology	Participants recognized technology’s role in supporting independent living for older adults. They discussed the challenges associated with aging, such as the risk of falling, and how technology can offer solutions for home safety and security.	*And also I suppose falling, maybe if there is a stairs in the house, at the top of the stairs, an alert that would come on, say if you were at the top of the stairs, be careful or something. It happened to me, that is why I am conscious of it.* [Older adult 01]
Adopting and using technology	Adoption and ongoing use of technology are hindered by a lack of familiarity and reluctance to use new devices. Participants expressed the need for gradual introduction of technology, appropriate training, and the importance of nonstigmatizing technology that adapts to their changing needs.	*I have a friend, she is older and she has the tablet she won’t even open it, even to do a text, it is easy, I will sit and show you. She is very intelligent and that but she has no interest.* [Older adult 02]

### Web-Based Survey and Web-Based Workshops

#### Web-Based Survey

The findings from the survey responses are reported elsewhere [19]. In brief, there was a high level of willingness reported across all groups (202/235, 86% older adults; 71/77, 91% family caregivers; 45/47, 96% health care professionals; and 16/21, 76% homecare staff) to use technology in the future to support older adults to live independently. Additionally, the analysis highlighted that key areas identified by older adult stakeholders where technology could assist in living independently were: home security (77/235, 33%), falls (69/235, 30%), reduced mobility (55/235, 23%), and loneliness (54/235, 23%). Thematic analysis of free text responses for other stakeholder groups highlighted that there were differences in which technology could best assist with independent living. The key areas that were identified were: remote monitoring of family members (family caregivers), communication with clients (health care professionals), and falls (homecare workers). The main disadvantages were considered to be the ability of some older adults to use the technologies, limited access to broadband, impaired cognition limiting the ability to use the technology, and the ability to interpret the data. Older adults perceived the main advantages of the technologies presented to be security or safety potential, the use of these devices to provide independence, and the ability to monitor their own health. Older adults reported the financial investment required and privacy concerns over data collected as the main disadvantages of these devices.

#### Web-Based Workshops

Participants of the web-based workshops discussed the potential value and concerns related to technology solutions identified by the technology partners. The findings are summarized in Table 2.

**Table 2.** Summary of technology types and participant value and concerns from the NEX web-based workshops.

Technology	Value for older adults	Concerns for older adults	Value for other stakeholders	Concerns for other stakeholders
Voice-activated assistant	Communication, entertainment, and activity	Dependency, privacy/intrusion, and data security	Reminders, social connections, entertainment, emergency calls, and promotes confidence	Dependence, disembodied voice, annoying accent, and issues for those with speech impediments
Ambient sensors	Confidence, falls prevention, security, and information for objective assessment	Intrusion/tracking and false alarms	Fall prevention, home security, emergency response activation, clinical benefits, person-centered monitoring, and value for caregivers	Maintenance, false alarms, complex data interpretation, and additional services required for monitoring
Wearables	Emergency response, falls detection, monitoring, and sleep	Stigma and forgetting to wear	Fall detection, reassurance, rehabilitation, and goal setting	Stigma, limited range (geofencing), cumbersome, aesthetically unpleasing, interferes with daily activities, and may forget to wear
Overall system	Reassurance and fosters independence	Trust issues, readiness, and burden on family	Favorable perception and substantial monitoring and information	Ability to adapt to different contexts and tailoring system to individual needs and preferences

### Prototype Testing

#### Overview

In the prototype testing, participants interacted with 4 types of technology: a wearable device (Sony mWatch), a voice-activated assistant (Amazon Echo 8), ambient sensors (Aeotec door and window sensor 6), and smart plugs (Samsung SmartThings smart plugs) installed in their home environment for a 10-week period. At the end of the testing phase, participants completed an evaluation interview. Although the small participant group size made it difficult to generalize the acceptability results, there appeared to be clear trends in the feedback from the prototype testing. The most salient findings from the thematic analysis of transcribed interviews in relation to users’ experiences of the prototype are outlined in Table 3 below.

**Table 3.** A summary of user experiences from prototype testing.

Technology	User feedback	Participant quotes
Wearable device	Participants were generally disappointed with the watch due to its large, chunky, and masculine design. They found it less attractive compared with other smartwatches like Fitbit, noted issues with its functionality including lack of step history, and frequent need for recharging.	*I didn’t like the watch, I didn’t like the style, it wasn’t a bit feminine.* [Participant 01] *I’ve seen watches that would give you yesterday’s and today’s steps but there was no history on this watch.* [Participant 04]
Voice-activated assistant	The response was mixed: some participants appreciated the device for its ability to provide companionship, access to music, and international radio stations. However, others viewed it as gimmicky and raised privacy concerns related to data sharing with Amazon.	*I felt it was a bit of company, to have a voice coming back at you…you could have a conversation with it...I just think it’s a wonderful piece of equipment. I live on my own.* [Participant 01] *If I was lonely, it was quite nice to have someone to talk to and who wouldn’t get cross with you.* [Participant 03]
Ambient sensors and Smart plugs	Participants quickly adapted to these technologies, noting a high level of comfort with their presence. Participants appreciated the sensors for their nonintrusive nature and the sense of security they provided, with minimal behavior modification observed.	*I forgot all about them, except occasionally I might notice the green light flashing if I opened the door.* [Participant 01] *… great to be able to know what state people are in and what they are doing without necessarily a camera being on them.* [Participant 03]

#### Post Design Analysis

To consider the implications of the co-design process on the final NEX system design, discussion workshops between the NEX team members (academic and industry project partners) were conducted. A public and patient involvement panel which was established at the outset of the project was also consulted at each stage of design to give expert opinion and advice on how to consider the findings at each iterative phase. The discussion workshops were based on (1) the findings of the pre- and co-design phases, and (2) an investigation of the technical performance of the system during prototype testing (Table 4).

**Table 4.** Key findings and recommendations from post design analysis phase.

Aspect	Key issues identified	Recommendations for future deployments
Participant experience	One participant withdrew due to the self-installation process.	Install the NEX system in participant’s homes by a technical expert.
Device use	Sony mWatch was found to be unsatisfactory.	Discontinue Sony mWatch and switch to an alternative wearable for data collection.
System performance	Battery life issues, configuration of sensors, system crashes, and missing data.	Implement automatic system data checks to reduce data losses.
Sensor configuration	Issues with battery life, participant-led installation, and devices turned off.	Review and use next-generation devices with longer battery life.
Data integrity	Missing data attributed to device and installation issues.	Use only pretested Smart Plugs and improve ground truth data collection.
Technical analysis	Front-end usability issues and back-end issues like memory use.	Deploy a larger range of sensors and smart plugs, and collect more frequent ground truth data.
ADL^a^ detection	Need for improved accuracy in ADL detection.	Deploy a broader range of sensors and smart plugs alongside more frequent ground truth data collection to enhance ADL detection accuracy.

a ADL: activities of daily living.

## Discussion

### Principal Findings

The results of this research have implications for researchers, practitioners, and digital health organizations who are aiming to design and implement technology-based solutions for health care. The co-design process used in this research facilitated the consideration of the needs and requirements of the proposed NEX system through a dynamic design process and design tool selection in response to a range of stakeholder perspectives.

### Barriers and Facilitators to Co-Designing Technology to Support Independent Living

In the development of the NEX system, a structured co-design methodology was used, incorporating a series of distinct phases that gathered and integrated insights from a diverse group of stakeholders. Initially, exploratory workshops and web-based surveys provided a broad array of perspectives from older adults, caregivers, health care professionals, and home care staff. These early phases captured essential needs and expectations, which were then analyzed in subsequent co-design sessions focused on refining the prototype. Discussion workshops between researchers and industry partners with input from a public and patient involvement panel were crucial for consolidating the findings from each phase. This process ensured that diverse insights were synthesized into a cohesive design strategy.

A systematic review [18] on co-designing technology for aging in place emphasizes the significant benefits of involving stakeholders in developing technological solutions for older adults, such as enhanced acceptance and adoption. However, the review also identifies that a lack of knowledge among participants can lead to unrealistic expectations, which may impede the design process. This knowledge gap poses a substantial barrier, particularly when co-designing with older adults who may not be familiar with the functionalities of smart devices. The challenge of addressing gaps in understanding the capabilities and benefits of technology was actively addressed during the NEX system’s co-design process. Due to COVID-19 constraints, at the early co-design stages (web-based focus groups and surveys) participants were not able to interact with some of the proposed technological solutions. To overcome this, technology images, descriptions of technology use, and demonstration videos were developed to showcase each of the potential technology solutions and their functionality. The images and text descriptions were embedded in the survey to assist with responses relating to the usefulness of technology and the demonstration videos were played via web-based focus groups to aid discussions.

### Technology Acceptability and Usability

The exploratory workshops and web-based surveys from the predesign phase underscored the critical importance of using technology to enhance the safety and security of older adults living independently. Interestingly, in the prototype testing, of all of the different technology types that make up the NEX system, ambient sensors were the most widely accepted technology among participants. Additionally, ambient sensors collected the most valuable data to assist with automated identification of ADL [24] which has been implicated as an important approach for supporting continued autonomous living for older adults in the future [25]. A smartwatch was also incorporated into the prototype design to address these needs with features like step count motivation for promoting a healthy lifestyle and emergency response capabilities for safety. However, the smartwatch was not well-received mainly due to its aesthetic features (interfered with clothing) and some technical issues with the GPS emergency response feature, rendering it less effective. Participants did appreciate the step count feature of the smartwatch. These findings prompted further exploration of alternative solutions for safety and security in the home environment for future iterations of the NEX system. Moore et al [26] note that older adults generally enjoy wearable devices that monitor steps, track location, log activities, measure health metrics, and offer automatic emergency contact features. However, continued use of devices requires more than just useful features; it necessitates a support system that motivates users, encourages social interaction, and adjusts to their preferences.

Consistent with the observations of Moore et al [26], the NEX system prototype testing revealed that research participants initially demonstrated high levels of engagement with new technology, but their engagement and potential interest can lessen over time [27]. This has been evidenced by others [28,29] who have investigated motivation among long-term users of assistive technologies. As part of the NEX project, the authors explored the intentions of older adults to adopt and use smart home technologies using the Theoretical Domains Framework, the results of which are reported elsewhere [30]. This work highlighted that unless methods to increase intrinsic motivation are considered in the design of such systems, long-term adherence is unlikely to be achieved. This is an important point of consideration for future research to promote consistent engagement by participants for the collection of data to support their well-being.

### Recommendations for Future Deployments

The results of the post design phase highlight the iterative nature of technology development in the context of IoT systems for older adults, emphasizing the need for continuous refinement and user-centered design to address practical challenges effectively. The withdrawal of a participant due to challenges with the self-installation process underscores the importance of user-friendly setup procedures. The recommendation for future deployments to include installation by a technical expert will ensure that participants are not burdened by technical complexities. Participants from the predesign phase also discussed the need for training and support to develop the digital literacy skills required to adopt technology to support independent living. Jiménez et al [31] outline the benefits of an iterative training approach for supporting older adults in the development of these skills. This approach involves a continuous process of training and support, allowing older adults to gradually build their digital literacy skills and confidence in using technology. This approach was subsequently implemented as part of the action research cycle which explored the feasibility and acceptability of NEX in a larger population of community-dwelling older adults. Another key observation from the prototype testing was the need for improved accuracy in detecting ADLs which is a critical aspect of the system’s functionality. The recommendation to deploy a broader range of sensors and collect more frequent ground truth data reflects an ongoing commitment to enhancing the precision and effectiveness of the system in real-world applications. Ghayvat et al [22] suggest larger numbers of sensor placements (approximately 30) for effective detection of ADLs in the homes of older adults however the acceptability of the placement of this number of sensors in the home environments of older adults needs to be explored further.

### Limitations

As for many other research projects, the COVID-19 pandemic and subsequent restrictions on movement limited the way the research teams could engage with participants and may have had implications for both co-design and prototype testing in this study. For aspects of the co-design process, focus groups were conducted via the web using Zoom as it was not possible to meet in person due to national COVID-19 guidelines. It is possible that richer discussions and opinions on how individual technology types may support independent living might have been achieved during in-person focus groups. Additionally, the prototype testing of NEX occurred during a time when restrictions on movement were in place and therefore the technician could not install the prototype in the homes of participants. Although participants self-installed the technology with the remote support of a technician, there were instances where technology failed (due to battery life) and data was lost, impacting the assessment of the technical performance of NEX. However, these adapted research approaches did facilitate the progression of this research during difficult circumstances.

### Conclusion

The co-design process described by the authors enabled the project team to use an agile approach and consider a range of stakeholders’ opinions in the design of this system. Although COVID-19 restrictions prevented face-to-face co-design research activities, the research team adapted research methods to facilitate web-based data collection and remote real-life testing. In terms of the research methodology, the authors presented an approach for a comprehensive co-design process involving older adults experiencing technology to support independent living over a sustained period in their own homes rather than conducting experiments and analyzing results based on limited exposure and assessment. The findings from this co-design process highlight that early participant engagement in the design process is necessary to ensure that the system meets the needs of stakeholders, which in turn supports technology adoption and cultivates motivation to use technology. An appreciation of the role of co-design and stakeholder opinions in terms of user needs and requirements by industry partners and clear and frequent communication channels were key attributes of a successful academic-industry collaboration in the area of digital health innovation.

## Abbreviations

ADL: activities of daily living
AI: artificial intelligence
DCU: Dublin City University
IoT: Internet of Things
